# Quarantine for pandemic influenza control at the borders of small island nations

**DOI:** 10.1186/1471-2334-9-27

**Published:** 2009-03-11

**Authors:** Hiroshi Nishiura, Nick Wilson, Michael G Baker

**Affiliations:** 1 Theoretical Epidemiology, University of Utrecht, 3584 CL Utrecht, the Netherlands; 2 Pandemic Influenza Research Group, University of Otago, Wellington, New Zealand

## Abstract

**Background:**

Although border quarantine is included in many influenza pandemic plans, detailed guidelines have yet to be formulated, including considerations for the optimal quarantine length. Motivated by the situation of small island nations, which will probably experience the introduction of pandemic influenza via just one airport, we examined the potential effectiveness of quarantine as a border control measure.

**Methods:**

Analysing the detailed epidemiologic characteristics of influenza, the effectiveness of quarantine at the borders of islands was modelled as the relative reduction of the risk of releasing infectious individuals into the community, explicitly accounting for the presence of asymptomatic infected individuals. The potential benefit of adding the use of rapid diagnostic testing to the quarantine process was also considered.

**Results:**

We predict that 95% and 99% effectiveness in preventing the release of infectious individuals into the community could be achieved with quarantine periods of longer than 4.7 and 8.6 days, respectively. If rapid diagnostic testing is combined with quarantine, the lengths of quarantine to achieve 95% and 99% effectiveness could be shortened to 2.6 and 5.7 days, respectively. Sensitivity analysis revealed that quarantine alone for 8.7 days or quarantine for 5.7 days combined with using rapid diagnostic testing could prevent secondary transmissions caused by the released infectious individuals for a plausible range of prevalence at the source country (up to 10%) and for a modest number of incoming travellers (up to 8000 individuals).

**Conclusion:**

Quarantine at the borders of island nations could contribute substantially to preventing the arrival of pandemic influenza (or at least delaying the arrival date). For small island nations we recommend consideration of quarantine alone for 9 days or quarantine for 6 days combined with using rapid diagnostic testing (if available).

## Background

Strict maritime quarantine (with facility quarantine on land in some cases), appeared to effectively prevent the entry of the 1918–19 influenza pandemic into American Samoa and delayed its entry into mainland Australia, Tasmania and New Caledonia [1]. Quarantine measures during this pandemic also worked successfully in Yerba Buena, an island off San Francisco [2], and within parts of Iceland [3]. More generally, a systematic review has reported evidence that interventions that included quarantine (2 studies) and isolation (10 studies) were effective in containing respiratory virus epidemics [4]. An earlier review had suggested a limited use for quarantine but had focused on quarantine attempts in countries with porous land borders [5].

Since it appears that quarantine was successful in island settings from 1918–19, some Pacific island nations have included the option of border quarantine in their current influenza pandemic plans [6]. Theoretically, since small island nations will most likely experience introduction of pandemic influenza at just one airport or seaport alone, the use of border control would be one of the most important options to protect their communities from the pandemic. As an example, New Zealand consists of multiple islands and has a pandemic plan that includes significant detail about border control and quarantine [6,7]. In addition, border quarantine is also included in the pandemic plans of some European countries [8].

However, detailed guidelines for effective use of quarantine have yet to be formulated. One of the key questions among infectious disease specialists and public health practitioners is how to optimise the duration of quarantine to achieve a desired level of effectiveness. Presently, there is no universal proposal for quarantine period following exposure to pandemic influenza cases. Although the etymological root of quarantine originates from 13th century public health practices requiring incoming ships to remain in port for 40 days [9], quarantine in the present day refers to compulsory physical separation for a defined period, including restriction of movement, of healthy individuals who have been potentially exposed to an infectious disease [10]. Since the restriction of movement often involves legal and ethical constraints, because it limits the freedom of quarantined individuals [11], the optimal length of quarantine needs to be clarified using scientifically sound approaches.

To suggest the optimal length of quarantine for pandemic influenza, we need to consider the detailed epidemiologic characteristics of this disease including the presence of asymptomatic infection [12]. The present study aimed to assess the potential effectiveness of quarantine, suggest an optimal length, and examine its potential performance for small island nations.

## Methods

### Hypothetical setting

To clarify the optimal length of quarantine, we first consider a hypothetical setting where infected travellers are flying from a nation with an epidemic (somewhere in Asia, given the data on the origin of seasonal influenza [13]) to a disease-free small island nation (e.g., New Zealand or smaller South Pacific and Caribbean islands). Specifically, we consider a situation when the disease-free country is fortunate enough to be informed about the possible emergence of the influenza pandemic at the source, sufficiently in advance of its arrival to implement border control measures. Given that the possible emergence is still uncertain and very recent news, we assume that the disease-free island nation is not ready or willing to completely shut down all its airports, but that quarantine is immediately instituted at the border. Before closing all the airports we assume that the island nation still permits the arrival of 20 aircraft with a total of 8000 incoming individuals (i.e., each with 400 individuals including airline staff on board) who were potentially exposed to influenza at the source country or on the aircraft. For this population of travellers we explore the question – how long should we place them in quarantine?

We assume that all incoming individuals are placed into routine quarantine on arrival in the island nation and are monitored for onset of symptoms during the quarantine period. We also assume that all infected individuals who develop influenza symptoms are successfully detected (e.g., through self-report questionnaires, reporting by ground staff, specific interview assessment by trained health personnel and/or thermal scanning). The impact of imperfect detection on the effectiveness of quarantine is examined in the Appendix. Optimistically, symptomatic cases are assumed to be immediately isolated in a designated facility at symptom onset, and assumed not to result in any secondary transmissions [14]. Similarly, those who developed symptoms en-route are also assumed to be successfully isolated upon arrival (and we ignore these individuals in the following analyses as the detection is owing to the entry screening). We assume that quarantine security would be fully effective and that no secondary transmission would occur in the quarantine facility. Successful detection during quarantine relies largely on onset of influenza-like symptoms, but, as a possible option, we also consider adding rapid diagnostic testing to improve the sensitivity of case detection.

### Epidemiologic characteristics of influenza

To theoretically and quantitatively examine the effectiveness of quarantine, we use several parameters describing the epidemiologic characteristics of seasonal influenza – which we then use for considering pandemic influenza. The most important of these characteristics is the cumulative distribution of the incubation period (i.e., the time from infection to onset) of length *t*, *F*(*t*). The incubation period has been very useful in suggesting the optimal length of quarantine for many diseases [15], because arbitrarily taking the 95th or 99th percentile point as the quarantine period could ensure the absence of symptomatic infection with probability of 95% or 99% [12,16-21]. However, it is difficult to directly apply this concept to influenza [12], because the conditional probability, *α*, of developing symptomatic disease (given infection) has been suggested to be 66.7% [22,23], and detection through quarantine is not relevant for asymptomatic infected individuals who account for the remaining 33.3%. Thus, we consider the effectiveness of quarantine as the reduction of the risk of introducing "infectious" individuals into the community and, thus, additionally use the cumulative distribution of the generation time (i.e., the time from infection of a primary case to infection of a secondary case by the primary case) of length *t*, *G*(*t*). Further, to simulate the key ripple benefit of quarantine (the predicted number of secondary transmissions caused by released infectious individuals), we assume that the reproduction numbers of symptomatic (*R*_s_) and asymptomatic cases (*R*_a_), i.e., the average numbers of secondary transmissions caused by a single symptomatic case and an asymptomatic case are 2.0 and 1.0, respectively. The basic reproduction number, *R*_0_, is therefore *αR*_s_+(1-*α*)*R*_a _= 1.67 which corresponds to an estimate in a previous study [24]. Moreover, the estimate is also within the estimated range of community transmission in another study which explored various historical data [25].

Distribution of the incubation period, which was assumed to follow a gamma distribution, was extracted from a published dataset [26]. Since the original data showed daily frequency of onset only, we fitted the cumulative distribution of the incubation period to the observed data, minimising the sum of squared errors. We did not identify more detailed data and note that the obtained frequency did not deviate much from outbreak data on an aircraft [27,28], a historical study of Spanish influenza [15,29], and from data in a published meta-analysis [22]. Similarly, the generation time was retrieved from a previous study of volunteers infected with influenza [22], which assumed that infectiousness is proportional to viral shedding, and we obtained the parameter estimates by minimising the sum of squared errors. A lognormal distribution was employed to model the generation time. Strictly speaking, the viral shedding curve alone does not inform the generation time, but our outcome measure (i.e., the probability of releasing infectious individuals) is reasonably analysed using virological data (as we are dealing with infectiousness), assuming that the frequency of contact is independent of time since infection. Furthermore, we favoured the use of this dataset as it would give a more conservative result since the right-tail is fatter than those assumed previously [30,31].

### Effectiveness of quarantine

Although secondary transmission on aircraft is probably relatively rare due to the functioning of ventilation systems [32,33], a previous transmission event has been reported in this setting [27]. Therefore, we use arrival time as the latest time of possible infection (i.e., *t *= 0). In other words, we conservatively argue the quarantine period as if all infected incoming individuals experienced this infection upon arrival. In reality, earlier acquisition of infection would increase the probability of non-infection after quarantine and therefore increase the effectiveness of quarantine. Although our worst case scenario potentially overestimates the optimal length of quarantine, a more realistic scenario requires the exact time of infection for all incoming infected individuals, which is in principle impractical (see Appendix for more detailed insights into this issue).

We considered the effectiveness of quarantine, *ε*(*t*), as a relative reduction of the risk of introducing infected individuals into the community as a function of time since infection *t*, i.e.,

(1) $$\varepsilon (t)=1-\frac{r_{1}(t)}{r_{0}(t)}$$

where *r*_1_(*t*) and *r*_0_(*t*) are the risks of releasing infected individuals into a new community in the presence and absence of the quarantine measure, respectively. Since all infected individuals enter the community without quarantine, we assume *r*_0_(*t*) = 1 for any *t*. If the risk in the presence of quarantine, *r*_1_(*t*), is regarded as the risk of releasing "symptomatic infected" individuals (regardless of infectiousness) after quarantine of length *t*, *r*_1_(*t*) is given by 1-*F*(*t*). Therefore, only the incubation period determines the effectiveness, i.e., *ε*(*t*) = *F*(*t*), which has been the fundamental concept in previous studies [12,15-21]. However, we further consider the infectiousness for influenza, emphasising the importance of asymptomatic infection, because the proportion 100×(1-*α*) is as large as 33.3%. We thus regard the risk *r*_1_(*t*) as the probability of releasing "infectious" individuals into the community after quarantine of *t *days.

To comprehensively discuss this issue we decompose *r*_1_(*t*) into the sum of symptomatic and asymptomatic individuals (denoted by *r*_1s_(*t*) and *r*_1a_(*t*), respectively). For those who will eventually develop symptoms, the probability of release, *r*_1s_(t), is

(2) *r*_1*s*_(*t*) = *α*(1 - *F*(*t*))(1 - *G*(*t*))

where *F*(*t*) and *G*(*t*) are, respectively, the cumulative distributions of the incubation period and generation time. Because of the absence of adequate data, we assume independence between the incubation period and generation time, which most likely yields conservative estimates of the effectiveness (compared to that explicitly addressing dependence between these two distributions). For those who remained asymptomatic throughout the entire course of infection, the probability *r*_1a_(t) is

(3) *r*_1*a*_(*t*) = (1-*α*)(1-*G*(*t*))

because the incubation period is not relevant to the detection of asymptomatic infected individuals. Due to the absence of data, it should be noted that we assume that the length of generation time among asymptomatic individuals is identical to that among symptomatic cases, an assumption that has been used by others [24,25]. As the assumption adds an uncertainty to the model prediction, we examine the potential impact of differing generation times between symptomatic and asymptomatic infected individuals (see Appendix). Consequently, the effectiveness of quarantine, *ε*(*t*), is given by subtracting *r*_1s_(*t*) and *r*_1a_(*t*) from 1: i.e.,

(4) *ε*(*t*) = 1 - [*α*(1 - *F*(*t*))(1 - *G*(*t*)) + (1 - *α*)(1 - *G*(*t*))]

We further investigate the additional benefit of testing for the pandemic influenza virus using rapid diagnostic testing during quarantine. A key assumption made is that the currently available diagnostic tests would perform as well with the new pandemic strain of virus (and be supplied to the islands in time). We assume that the sensitivity (S_e_) and specificity (S_p_) of the rapid diagnostic test are 69.0% and 99.0%, respectively [34]. Since our effectiveness measure is conditioned on infected individuals, the risk of releasing infectious individuals in the presence of quarantine with use of rapid diagnostic testing is obtained by multiplying a factor (1-S_e_) to *r*_1_(t) which represents a proportion of cases that are missed even following rapid diagnostic testing. Thus, we get the effectiveness *ε*_d_(*t*) as

(5) *ε*_*d*_(*t*) = 1 - (1 - S_e_) [*α*(1 - *F*(*t*)) (1 - *G*(*t*)) + (1 - *α*) (1 - *G*(*t*))]

Due to the absence of more detailed data, we assume that both the sensitivity and specificity of the rapid diagnostic test are independent of time since infection. Considering that the sensitivity may well decline in later stages of illness (by implicitly assuming that the diagnostic test is correlated with viral load), it should be noted that the results associated with equation (5) are probably most valid only for those in the early stage of illness (which is consistent with our particular interest in quarantine period). We stress that the estimated effectiveness *ε*_d_(*t*) for a long quarantine period (e.g., longer than 8 days) should be treated cautiously. Since the sensitivity S_e _of asymptomatic infected individuals may be smaller than that among symptomatic cases (due to lower virus shedding titres among asymptomatic individuals), we examine the effectiveness of quarantine with differing S_e _between symptomatic and asymptomatic infected individuals (see Appendix).

### Sensitivity analysis and preventive performance

We also examined the sensitivity of our effectiveness measures (4) and (5) to different lengths of quarantine and prevalence levels at the source by means of simulations. First, the sensitivity was assessed using the number of released infectious individuals after quarantine of length *t*. We examined plausible prevalence levels of 1%, 5% and 10% at the source, which respectively indicate that there were 80, 400 and 800 infected individuals among a total of 8000 incoming individuals. The highest prevalence, 10%, may represent transmission events within an airport of the country of origin or on an aircraft. The analysis was made by randomly simulating the incubation period (*F*), the generation time (*G*), the presence of any symptoms (*α*) and the sensitivity of the rapid diagnostic test (S_e_) where *F *and *G *randomly follow the assumed gamma and lognormal distributions, respectively. The two dichotomous variables (i.e., the presence of symptoms and sensitivity of the rapid diagnostic test) were randomly simulated with uniform distributions (i.e., drawing random real numbers from 0 to 1) and using cut-off points at *α *= 0.667 and S_e _= 0.690. The random sampling was performed for the number of infected individuals (80, 400 and 800 times) in each simulation, and the simulation was run 100 times for each length of quarantine and prevalence level. To show the ripple benefit, we also investigated the number of secondary transmissions caused by released infectious individuals. This estimate was achieved by further randomly simulating the numbers of symptomatic and asymptomatic secondary transmissions. Both numbers were assumed to follow Poisson distributions with mean *R*_s_(1-*G*(*t*_d_))(1-*F*(*t*_d_)) and *R*_a_(1-*G*(*t*_d_)), respectively, for each of the released symptomatic and asymptomatic infectious individuals after the quarantine of length *t*_d _days.

**Figure 1:**
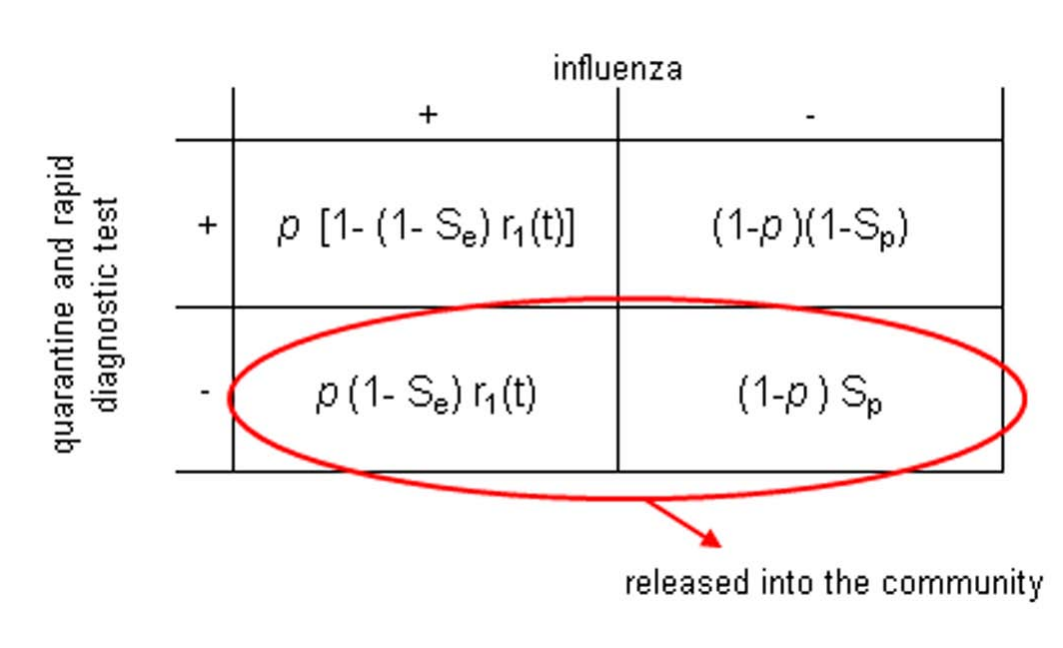
**Performance of quarantine combined with rapid diagnostic testing**. *r*_1_(*t*) is the probability of releasing infectious individuals following the quarantine of length *t *days. S_e _= sensitivity of the rapid diagnostic test; S_p _= specificity of the rapid diagnostic test; *p *= prevalence at the source community. Among infected individuals, those testing negative after quarantine of length *t *(i.e., *p*(1-S_e_)*r*_1_(t)) are released into the community. Among uninfected individuals, those testing negative (i.e., (1-*p*)S_p_) are released.

Finally, we examined the preventive performance of quarantine combined with rapid diagnostic testing. When the combination scheme is employed, those testing negative to the rapid diagnostic test following quarantine of length *t *would be the population of interest, as they are then released into the community. Let *p *be the prevalence level at the source (0 ≤ *p *≤ 1). Among infected individuals (who account for 100*p*% of the travellers), the fraction of those who are detected or lose infectiousness following quarantine of length *t *(i.e., true positives) is (1-*r*_1_(t)). Of the remaining infected individuals *r*_1_(t), the fraction of those testing positive, S_e_*r*_1_(t), to the rapid diagnostic test are placed into isolation and, thus, are added to the true positives. Consequently, the remaining fraction (1-S_e_)*r*_1_(t) are false negatives and are released into the community (Figure 1). Among uninfected individuals (i.e., 100(1-*p*)% of the travellers), the length of quarantine does not influence the preventive performance (because they are not infected and their quarantine is irrelevant to the loss of infectiousness). Thus, among the total number of incoming travellers, the fractions (1-*p*)(1-S_p_) and (1-*p*)S_p _will be testing positive (false positives) and negative (true negatives), respectively, to the rapid diagnostic test. Consequently, positive predictive value (PPV) of quarantine combined with rapid diagnostic testing is

(6) $$\text{PPV}=\frac{p[1-(1-{\text{S}}_{\text{e}})r_{1}(t)]}{p[1-(1-{\text{S}}_{\text{e}})r_{1}(t)]+(1-p)(1-{\text{S}}_{\text{p}})}$$

whereas negative predictive value (NPV) is

(7) $$\text{NPV}=\frac{(1-p){\text{S}}_{\text{p}}}{p(1-{\text{S}}_{\text{e}})r_{1}(t)+(1-p){\text{S}}_{\text{p}}}$$

PPV measures the preventive performance of quarantine policy to correctly place infected individuals in quarantine (or isolation) during their infectious period (i.e., how efficiently are we placing infectious individuals in the quarantine facility, among a total of those who are diagnosed as positive either by quarantine of length *t *or rapid diagnostic testing). NPV measures the preventive performance of the release policy (i.e., how large is the fraction of true negatives among a total of those who are diagnosed as negative after the quarantine of length *t *and rapid diagnostic testing). We numerically computed both PPV and NPV for different prevalence levels (from 0–15%) and different lengths of quarantine (from 0 to 10 days). All analyses were made using the statistical software JMP ver. 7.0 (SAS Institute Inc., Cary, NC).

## Results

### Intrinsic dynamics of influenza

**Figure 2:**
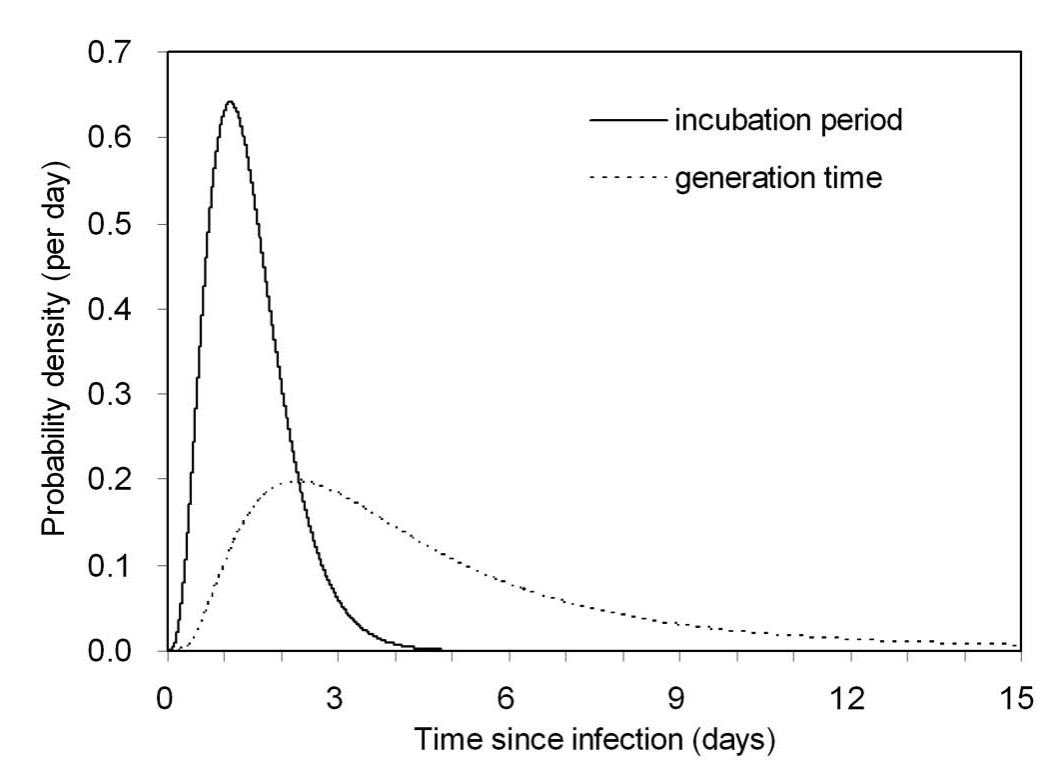
**Probability density functions of the incubation period and generation time of influenza**. Gamma distribution was employed to model the incubation period (i.e., the time from infection to onset), whereas lognormal distribution was fitted to the generation time (i.e., the time from infection of a primary case to infection of a secondary case by the primary case). The mean and variance of the incubation period and generation time are estimated as 1.43 days and 0.48 days^2 ^and 2.92 days and 5.57 days^2^, respectively. For the original data see: [22] and [26].

Figure 2 shows density functions of the incubation period and generation time. The incubation period was similar to those reported previously [12,27,28]. Mean and variance of the incubation period were estimated as 1.43 days and 0.48 days^2^, respectively. The generation time in the figure includes the original datasets on different types of influenza virus (weighed by each sample size). The mean, median (25–75th percentile) and variance of the generation time were 2.92 days, 2.27 (1.41–3.67) days and 5.57 days^2^, respectively.

### Effectiveness of quarantine

**Figure 3:**
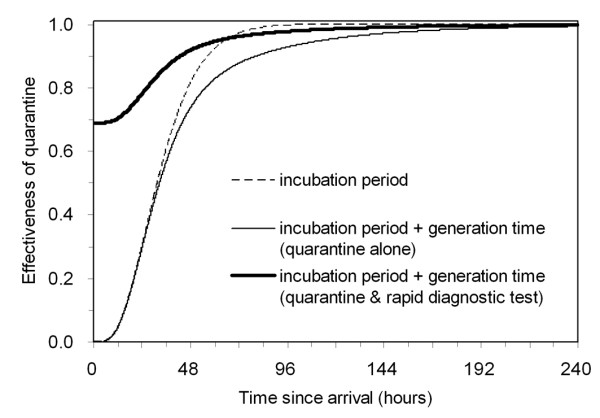
**Effectiveness of quarantine with and without use of rapid diagnostic testing as a function of time since infection (i.e., time since arrival)**. Different effectiveness measures of quarantine are comparatively shown. The dashed line represents the effectiveness of quarantine, measured as the relative reduction of the risk of releasing "symptomatic infected" individuals (regardless of infectiousness) based on the incubation period alone. The two continuous lines measure the effectiveness as the relative reduction of the risk of releasing "infectious individuals" into the community, based on the incubation period, generation time and probability of symptomatic disease, with (thin) and without (thick) use of rapid diagnostic testing. The sensitivity of the rapid diagnostic test was assumed to be 69.0% (based on current test performance for seasonal influenza A [34]).

Figure 3 shows the estimated effectiveness of quarantine as a function of time since infection (i.e., time since arrival). A different effectiveness measure (i.e., relative reduction of the risk of releasing "symptomatic infected" individuals regardless of infectiousness) is shown (dashed line) comparatively with the other two results showing the relative reduction of the risk of releasing "infectious" individuals in the presence and absence of the use of rapid diagnostic testing (thin and thick solid lines, respectively). It should be noted that the reduction of symptomatic infected individuals is based only on the incubation period, measuring a different concept of effectiveness from other two. The incubation period alone suggests that 95% effectiveness in preventing the release of symptomatic infected individuals is achieved by quarantine of 2.73 days.

We predict that 95% and 99% effectiveness in preventing the release of infectious individuals is achieved with quarantine periods of longer than 4.74 and 8.62 days, respectively. As can be observed from Figure 3, the impact of using rapid diagnostic testing on effectiveness is larger for a short quarantine period. If a rapid diagnostic test was available and this performed to the current standard for detecting influenza A in the pre-pandemic setting, we estimated that this additional testing would result in quarantine periods for longer than 2.59 and 5.71 days having effectiveness of over 95% and 99% respectively (Figure 3).

### Sensitivity analysis

Given the above mentioned results, we investigated the sensitivity of quarantine effectiveness to four different lengths of quarantine (2.8, 4.8, 5.7 and 8.7 days) and to three different prevalence levels at the source (1%, 5% and 10%). The shortest length, 2.8 days, was suggested by the incubation period as being 95% effective in preventing the release of symptomatic infected individuals into the community. Two of the others (4.8 and 8.7 days) corresponded to 95% and 99% effectiveness in preventing release of infectious individuals by means of quarantine alone, and 5.7 days corresponded to 99% effectiveness when quarantine was combined with rapid diagnostic testing.

**Figure 4:**
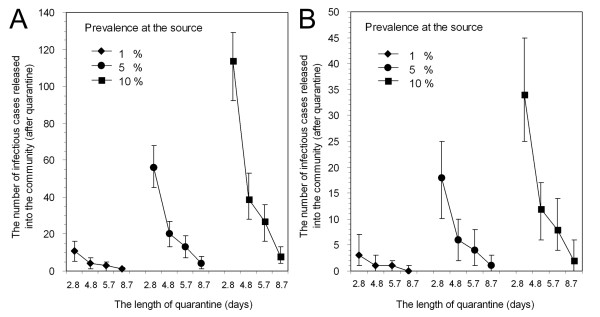
**Sensitivity of the number of infectious cases released into the community (after quarantine) to the different lengths of quarantine and prevalence levels at the source**. **A**. Quarantine alone. **B**. Quarantine combined with rapid diagnostic testing. Sensitivity of the number of released infectious cases into the community (after quarantine) is examined for different lengths of quarantine (2.8, 4.8, 5.7 and 8.7 days) and prevalence levels at the source (1%, 5% and 10%). Each dot represents median estimate of 100 simulation runs. The whiskers extend out to 5th and 95th percentiles of the simulations.

Figure 4 shows the median (and 5–95th percentile) numbers of infectious individuals who are released into the community after quarantine of specified lengths. The quarantine for 2.8 days could miss as many as 11 (5–16), 56 (45–68) and 114 (92–129) infectious cases for the prevalence of 1%, 5% and 10%, respectively in the 8000 arriving travellers considered. However, these misses were reduced to 4 (1–7), 20 (13–27) and 39 (28–53) cases by the quarantine of length 4.8 days, to 3 (0–5), 13 (7–19) and 27 (16–36) by 5.7 days and, moreover, to 1 (0–2), 4 (1–8) and 8 (4–13) cases by 8.7 days. The additional diagnostic testing could greatly reduce the released number of infectious individuals (Figure 4B). For the quarantine lengths of 2.8 and 5.7 days with rapid diagnostic testing, 3 (1–7), 18 (10–25) and 34 (25–45) cases and 1 (0–2), 4 (1–8) and 8 (4–14) cases, respectively, were expected be released into the community for the prevalence of 1%, 5% and 10%. All values for the quarantine period of 5.7 days combined with use of a diagnostic test were less than 3% of the total number of incoming infected individuals.

**Figure 5:**
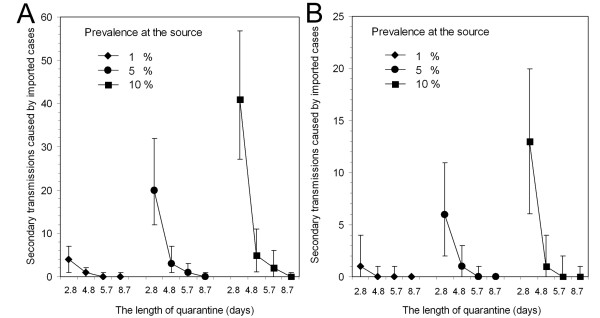
**Sensitivity of the number of secondary transmissions caused by released infectious individuals to the different lengths of quarantine and prevalence levels at the source**. **A**. Quarantine alone. **B**. Quarantine combined with rapid diagnostic testing. Sensitivity of the number of secondary transmissions caused by released infectious individuals is examined for different lengths of quarantine (2.8, 4.8, 5.7 and 8.7 days) and prevalence levels at the source (1%, 5% and 10%). Each dot represents median estimate of 100 simulation runs. The whiskers extend out to 5th and 95th percentiles of the simulations.

Figure 5 describes the ripple benefit of quarantine, expressed as the number of secondary transmissions caused by released infectious individuals. The qualitative patterns found were similar to those of Figure 4, but it should be noted that no secondary transmission was observed in the community in several scenarios. Quarantine of length 2.8 days, with or without rapid diagnostic testing, would lead to many secondary transmissions caused by released infectious individuals. When there was quarantine of 4.8 days without rapid diagnostic testing, we found 0 (0–2), 3 (1–7) and 5 (1–11) secondary transmissions. Extending quarantine to 8.7 days resulted in no secondary transmissions at prevalence levels of 1%, 5% and 10% (i.e., all were 0 except for 1 secondary transmission at the 95th percentile for all three prevalence levels). When diagnostic testing was combined with the quarantine period for 5.7 days, 0 (0–1), 0 (0–1) and 0 (0–2) secondary transmissions resulted. That is, even though quarantine alone for 8.7 days and quarantine combined with diagnostic testing for 5.7 days permit the release of several infectious individuals (up to 3% of the total number of incoming infected passengers), the majority of the released cases are at the late stage of infection and hardly cause secondary transmissions in the island nation (i.e. even in the worst case, only a few secondary transmissions would be expected).

### Preventive performance of quarantine with rapid diagnostic testing

**Figure 6:**
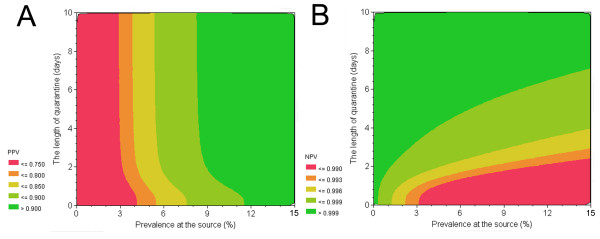
**Diagnostic performance of quarantine with use of rapid diagnostic testing**. **A**. Positive predictive values (PPV) and **B**. Negative predictive values (NPV) of quarantine combined with rapid diagnostic testing as functions of the length of quarantine and prevalence at the source. For the quarantine of 3 days or longer, PPV is less sensitive to the length of quarantine and depends almost only on the prevalence. NPV is sensitive to both the length of quarantine and prevalence at the source, achieving extremely high estimates to correctly release true negative individuals into the community.

Figure 6 shows contour plots of PPV and NPV of quarantine combined with rapid diagnostic testing as functions of the length of quarantine and prevalence of influenza at the source. Given a fixed prevalence, PPV was greater for shorter length of quarantine (especially, for *t *< 1 day) due mainly to the relative increase in detection of true positives by rapid diagnostic testing (see equation (6)). However, it became less sensitive to the length of quarantine as the length became longer (for *t *> 2 days) and depended almost only on the prevalence (Figure 6A). Figure 6B demonstrates that NPV was on the whole very high and sensitive to both the length of quarantine and prevalence at the source. For prevalence levels up to 10%, NPV with quarantine for longer than 2 days could be greater than 99.0%. In particular, at a quarantine length of 6 days, NPV was greater than 99.9% for prevalence levels up to 10%. In other words, within the range of interest for quarantine lengths, PPV was mainly determined by the prevalence level (i.e., longer quarantine with rapid diagnostic testing does not load too many additional false positives on isolation facilities compared with the use of shorter quarantine and testing). Also, NPV can be extremely high, indicating that the release policy can efficiently suggest that the released individuals are likely to be true negatives.

## Discussion

The present study provides theoretical support for border quarantine as a worthwhile pandemic influenza control measure for small island nations. Detailed advance planning for quarantine measures may therefore be justified during the pre-pandemic period. From our quantitative findings, we recommend a quarantine period of 9 days (rounding 8.7 days to the next integer) to reduce by more than 99% the risk of introducing infectious individuals and to ensure the absence of secondary transmissions caused by released infectious individuals in the community. If the use of rapid diagnostic testing can be combined with quarantine, the quarantine period could be shortened to 6 days (rounding 5.7 days to the next integer). To the best of our knowledge, the present study is the first to explicitly suggest an optimal length of quarantine for pandemic influenza derived from detailed epidemiologic characteristics of this infection. Although our recommendations are based on arbitrarily considering the specific percentiles of effectiveness, and although the absence of secondary transmissions depends also on the absolute number of incoming individuals, we believe that our findings (with realistic ranges of prevalence and the number of travellers) provide evidence-based estimates that can be used for pandemic planning. Quarantine might ultimately be unsuccessful in preventing importation of infected individuals [35]. However, delayed entry of the pandemic virus could provide time to introduce other social distancing and pharmaceutical interventions that may reduce the overall impact of a pandemic [1,9,30,36-39].

In recent studies, the optimal length of quarantine was considered by using the incubation period distribution alone, identifying the 95th or 99th percentile point of the theoretical distribution [16-21]. For instance, 95th percentiles of the incubation period for severe acute respiratory syndrome (SARS) and smallpox were suggested to be 11–13 days [19,21] and 16–17 days [16] since exposure, respectively. Direct application of this concept to pandemic influenza suggests that the optimal quarantine period for pandemic influenza is only 2.73 days since exposure, which is far shorter than those for SARS and smallpox. However, since influenza involves a non-negligible fraction of asymptomatic infections [12,22], we also undertook the additional step of incorporating this feature into our assessment of quarantine effectiveness. This refinement permitted further elaboration of effectiveness estimates, which we believe contributes to theoretical considerations around the control of other infectious diseases. In addition, we reasonably showed the preventive performance of quarantine, expressed as the number of released infectious individuals and the ripple benefit expressed as number of secondary transmissions caused by them. Using further information on the contact structure in the island nation, our framework could be further extended to estimate the probability of extinction and the delay effect of epidemic spread imposed by quarantine, the latter of which was discussed by a recent study [35]. Although the recent study theoretically emphasises the difficulty of effective border control (including quarantine) [35], we stress that the epidemiologic characteristics of influenza (e.g., short incubation period and generation time) permit anticipating large ripple benefits from quarantine (given that importation may continue for only a short period of time before full border closure occurs).

Access to a highly sensitive test for pandemic influenza infection may increase the effectiveness of quarantine and shorten the quarantine period routinely required for incoming travellers. Preventive performance in finding true positives (i.e., PPV of quarantine combined with rapid diagnostic testing) appeared not to be very sensitive to the length of quarantine (for *t *> 2 days). This result suggests that if diagnostic test kit supplies are plentiful, then testing should be done early in quarantine. But if a test kit sparing approach is used (i.e., avoiding testing of those who become symptomatic) then there is not much benefit in delaying testing until after day 2 in quarantine. A test with both high sensitivity and high specificity would also allow for better use of resources if the travellers who tested negative are released into the community. Since PPV is mainly determined by prevalence at the source, it should be noted that an exit screening process at the source lowers the prevalence as well as PPV. Nevertheless, the effectiveness of quarantine itself is independent of the prevalence, and moreover, lower prevalence among incoming individuals yields a higher chance of extinction (or greater ripple effect of quarantine (Figures 4 and 5)). NPV of quarantine combined with diagnostic testing would be extremely high with quarantine periods for lengths of 3 days or longer, supporting our suggestion to release quarantined individuals testing negative to the rapid diagnostic test into the community (if there was high confidence in test performance parameters for the emergent pandemic strain). In light of our findings, island countries may consider including influenza testing capacity and test kit stockpiles in their pandemic plans. The use of rapid diagnostic tests, if available through stockpiling in advance or rapid delivery after pandemic emergence, may permit more effective border control, with more efficient use of isolation facilities and shortening of the quarantine period.

The operation of quarantine would be most feasible for islands with low traveller numbers and with pre-existing facilities that could be used for quarantine (e.g., hotels). Our study was indeed motivated by the consideration of protecting small island nations (e.g., in the South Pacific and Caribbean), because use of border control at usually just one or two international airports would be the major way in which the introduction of pandemic influenza could be prevented in these islands. Yet the analysis could potentially hold for larger island nations such as Australia, whose pandemic plan also includes border quarantine [40]. The logistics of quarantine might be far more demanding in Australia with its multiple international airports, but which nonetheless used strict maritime quarantine to successfully delay the entry of the 1918 pandemic [41]. Evidence about the geographic spread of influenza highlights the importance of quarantine in multiple locations [42-44]. Small countries with land borders and limited entry points could also use these approaches to delay entry of pandemic influenza as occurred for Israel in the 1957 influenza pandemic [5]. Facility-based quarantine could also be supplemented with ongoing surveillance in the community of those released from quarantine.

Our analysis employed a number of simplifying assumptions, among which we should emphasise the most important one. The detailed natural history parameters for seasonal influenza are not well documented and, moreover, we of course do not know if the incubation period and generation time of an emergent pandemic strain would be close to those of seasonal influenza documented in the limited number of publications to date. It should be noted that our analysis is solely based on the available published evidence and that the effectiveness of quarantine would be overestimated if the emergent strain of pandemic influenza had a longer incubation period or a longer generation time than we have assumed. However, the incubation period for human infection with H5N1 appears to be similar to other sub-types infecting humans [45]. This issue applies not only to the incubation period but also to other parameters, the role of which for each can be inspected using equations (4) and (5). For example, a historical analysis suggests that only 9% of infections resulted in an asymptomatic infection [46], which would contribute to improved quarantine effectiveness (compared to our results). Given that our exercise indicates the critical importance of the incubation period and generation time, epidemiological investigations should be performed to better quantify these parameters and further inform evidence-based pandemic planning.

Extrinsic factors should also be more precisely quantified in future. As an indirect extrinsic effect, when infected individuals are released into the community and become infectious to others, recently quarantined individuals may be detected and isolated earlier than those who have not been quarantined [47]. Another issue of detection is that some island states may have access to laboratory-based PCR influenza tests which are far more sensitive and specific than rapid tests [48], which could offer the test results in a few hours and greatly shorten the length of quarantine.

To more appropriately quantify the effectiveness of quarantine, two other technical issues have to be discussed. The first is concerned with skewness of the offspring distribution (i.e. the distribution of the number of secondary transmissions caused by a single primary case). Although our study reasonably showed the absence of secondary transmissions for quarantine of certain lengths, we ignored the skewness (i.e., the presence of potential super-spreaders [49]), and thus, the uncertainty bounds might have been smaller than in reality. Although the mean and median of the predicted number of secondary transmissions are still valid, and even though the skewed offspring distribution was partly incorporated in the model with the right-skewed generation time distribution, super-spreading events played a key role in triggering the international spread during the epidemic of SARS, and in light of this, quantification of the dispersion parameter (of the offspring distribution) is needed in future studies. Another issue is related to our conservative assumption that all incoming individuals experienced infection upon arrival. Since it is impractical to know the time of infection for all incoming infected individuals (which should ideally be known when the quarantine is started at time *t *= 0), we adopted a worst case scenario where all infected individuals experience infection at *t *= 0 (see Appendix). This assumption could have overestimated the optimal length of quarantine. If further research demonstrates that influenza transmission on board flights is very rare, then it would be possible to set the quarantine period to begin at the start of the flight and therefore reduce its duration correspondingly following arrival. However, then we have to take into account the possible secondary transmissions during the quarantine period. Estimation of the effectiveness of imperfect quarantine (i.e., quarantine which allows secondary transmissions within the quarantine facility) would be far more complicated than our simpler model, and clarification on this point is a task for future research.

In addition to the present study, it should be noted that quarantine may be combined with reduction of travel volumes (e.g., even mandatory restrictions on non-essential travel) which would have a large effect if it occurred rapidly [35,50,51]. Substantial reductions of travel volumes could make the logistics of quarantine far more feasible for island nations and increase the probability of ensuring the absence of secondary transmissions (given the same prevalence level to that of a larger travel volume). Moreover, there is the potential usefulness of antiviral prophylaxis during the quarantine period which could theoretically reduce the number of infectious individuals. Despite the plausible reduction of infectiousness under antiviral prophylaxis, the probability of symptomatic infection will also likely be reduced, and thus, the detection of cases might be reduced. Unless the efficacy of antiviral prophylaxis and detection under this measure are well documented and promisingly high, it is difficult to determine if this countermeasure is likely to offer an overall positive impact on the success of quarantine, and this point should be clarified in future research. Another topic area to be clarified further is concerned with cost-effectiveness. Although we implicitly assumed that the governments of island nations may be willing to allocate quarantine facilities and spend sufficient money for diagnostic testing, these measures are economically demanding, especially for developing island nations. Extension of our method would permit estimating the required cost to achieve a specific ripple benefit (e.g., zero secondary cases for a certain period of time). Use of home-based quarantine (with health agency surveillance and support) is another cost-saving option that could be considered for islands with limited capacity for using facility-based quarantine (e.g., those with few hotels that could be requisitioned), but it should be noted that home-based quarantine might violate our assumption of ignoring secondary transmissions during the quarantine period. In practice, there may also be scenarios where it is not practical to separate all incoming travellers into separate quarters within a quarantine facility (e.g. parents with small children). In such cases, health workers may need to monitor such individuals especially closely and isolation may need to include a parent and infant when only one is symptomatic (all of which would increase costs).

Despite our simplifying assumptions, the present study reasonably suggests that use of quarantine has the potential to substantially reduce the risk of pandemic influenza arriving or at least significantly delay arrival, in small island nations. To ensure the absence of secondary transmissions for plausible ranges of prevalence at the source and a modest number of incoming travellers, we recommend quarantining the incoming individuals for 9 days if quarantine alone is implemented and 6 days if quarantine is combined with rapid diagnostic testing.

## Conclusion

To inform border control for pandemic influenza in small island nations we examined the potential effectiveness of quarantine using several parameters which describe the epidemiologic characteristics of influenza. In particular, our modelling approach accounted for asymptomatic infection which is deemed a key requirement for successful influenza control [52,53]. The effectiveness was modelled as a relative reduction of the risk of introducing infectious individuals into the community as a function of time since arrival. We recommend a quarantine period of 9 days to reduce by more than 99% the risk of introducing infectious individuals and to ensure the absence of secondary transmissions. When rapid diagnostic testing is combined with quarantine, we recommend quarantine for 6 days to similarly prevent secondary transmissions.

## Competing interests

The authors declare that they have no competing interests.

## Authors' contributions

NW and MGB conceived of the study and participated in its design and coordination. HN developed methodological ideas and performed statistical analyses. HN and NW did most of the work on drafting the manuscript. All authors read and approved the final manuscript.

## Appendix

### Earlier infection before quarantine

For simplicity, we consider the impact of earlier exposure to infection on the effectiveness of quarantine in terms of the frequency of onset during the quarantine period, which is relevant to the determination of the incubation period conducted by Anderson Grey McKendrick [15,29]. Let the length of quarantine be *t*. To account for earlier infections before starting quarantine at *t *= 0, we consider infection-age (i.e. the time since infection) for infected individuals, denoted by *τ*. Let *i*(*t*, *τ*) and *j*(*τ*), respectively, be the number of incubating infected individuals at quarantine period *t *and infection-age *τ *and the number of incubating infected individuals at infection-age *τ *at the beginning of quarantine *t *= 0 (i.e. *i*(0, *τ*) = *j*(*τ*)). *i*(*t*, *τ*) is written as

(A1) $$i(t,\tau )=j(\tau -t)\frac{\Gamma (\tau )}{\Gamma (\tau -t)}$$

for *τ *-*t *> 0 where Γ (*τ*) informs the survivorship function of incubating individuals at infection-age *τ*, i.e.,

(A2) $$\Gamma (\tau )=\exp\left(-\int_{0}^{\tau }\gamma (\sigma )d\sigma \right)$$

where *γ *(*τ*) is the rate (or force) of onset at infection-age *τ*. Consequently, the density function of the incubation period, *f*(*τ*), is given by

(A3) *f*(*τ*) = *γ*(*τ*)Γ(*τ*)

Since we assume that there is no secondary transmission during quarantine period, *i*(*t*, *τ*) = 0 for *t*-*τ *> 0. The number of new symptomatic cases at quarantine of length *t*, *n*(*t*), is

(A4) $$n(t)=\int_{t}^{\infty }\gamma (\tau )i(t,\tau )d\tau$$

Replacing the right-hand side of (A4) by that of (A1), we get

(A5) $$n(t)=\int_{t}^{\infty }\gamma (\tau )j(\tau -t)\frac{\Gamma (\tau )}{\Gamma (\tau -t)}d\tau =\int_{0}^{\infty }f(t+\sigma )\frac{j(\sigma )}{\Gamma (\sigma )}d\sigma$$

In our setting, all quarantined individuals have not experienced symptom onset before quarantine starts at *t *= 0. Assuming that all infected individuals eventually experience symptom onset (just for now), the total number of infected individuals satisfies

(A6) $$\int_{0}^{\infty }n(t)dt=\int_{0}^{\infty }j(\tau )d\tau$$

Using (A5) and (A6), the density of symptom onset at quarantine period *t *(i.e. the frequency of symptom onset relative to the quarantine period *t*), *h*(*t*), is

(A7) $$h(t)=\frac{n(t)}{\int_{0}^{\infty }n(t)dt}=\int_{0}^{\infty }\frac{f(t+\sigma )}{\Gamma (\sigma )}\frac{j(\sigma )}{\int_{0}^{\infty }j(s)ds}d\sigma$$

Equation (A7) indicates the critical importance in understanding the earlier exposure in order to determine the optimal length of quarantine. That is, the density of symptom onset *h*(*t*) always depends on the infection-age distribution (which is informed by *j*(*τ*)) at the starting time point of quarantine (*t *= 0).

If the epidemic at the source country becomes endemic and reaches a stationary state with constant incidence *Q*, and if the infected travellers result from random sampling of infected individuals at the source country, we have *j*(*τ*) = *Q*Γ(*τ*), leading to

(A8) $$h(t)=\frac{\Gamma (t)}{\int_{0}^{\infty }\Gamma (\tau )d\tau }$$

which is equivalent to the survivorship of the incubating infected individuals (written as 1-*F*(*t*) in the main text using the cumulative distribution function of the incubation period *F*(*t*)). The simplification in (A8) holds only when a stationary state is the case at the source country, which is not likely to be observed in the event of an influenza pandemic. Thus, we need to use (A7) with some prior information of *j*(*τ*). Nevertheless, since the infection event is unobservable, we seldom know *j*(*τ*). Therefore, we recommend assuming that the start of quarantine *t *= 0 as the time of infection, which is the worst case scenario. Although the above mentioned arguments apply to symptomatic cases alone, we find exactly the same issue in the survivorship of infectiousness.

### Differing parameters between symptomatic and asymptomatic cases

First, we consider the impact of differing generation times between symptomatic and asymptomatic cases on the effectiveness of quarantine. Although the generation time distribution of asymptomatic influenza infection has yet to be clarified, we at least theoretically separate the cumulative distributions *G*_s_(t) and *G*_a_(t), respectively, for symptomatic and asymptomatic cases. The equation (4) in the main text is replaced by

(A9) *ε*(*t*) = 1 - [*α*(1 - *F*(*t*))(1 - *G*_s_(*t*)) + (1 - *α*) (1 - *G*_*a*_(*t*))]

Since *G*_a_(t) is unknown, we examine the sensitivity of *ε *(*t*_*β*_), where the effectiveness is calculated as 100*β *% (i.e. *β *= 0.95 and 0.99), to different ratios of *G*_a_(*t*_*β*_) to *G*_s_(*t*_*β*_). Let *c *be *G*_a_(*t*_*β*_)/*G*_s_(*t*_*β*_). *G*_s_(*t*) is assumed to be equivalent to *G*(*t*) in the main text.

**Figure 7:**
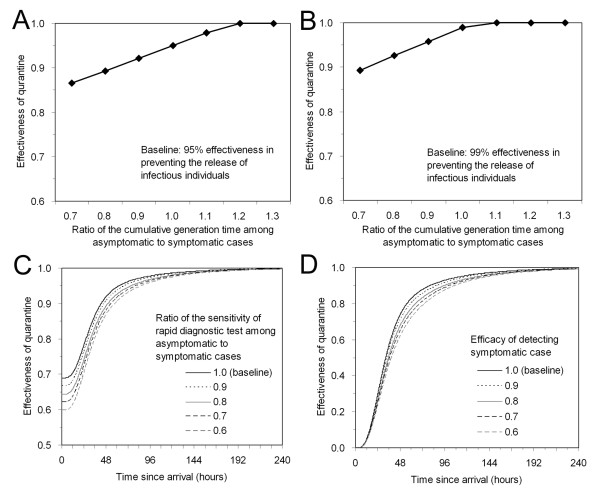
**Sensitivity of the effectiveness of quarantine to uncertain epidemiologic variables**. A & B. Effectiveness of quarantine as a function of the ratio of the cumulative generation time among asymptomatic to symptomatic cases. Sensitivity of the point estimates of the effectiveness with baseline values of 95% and 99% are examined in A and B, respectively. C. Sensitivity of the effectiveness of quarantine in the presence of rapid diagnostic testing to the diagnostic sensitivity among asymptomatic infected individuals. D. Effectiveness of quarantine with imperfect efficacy of case detection of symptomatic cases.

Figures 7A and 7B show the sensitivity of *ε *(*t*_*β*_) to different values of the ratio *c *with *t*_*β *_= 4.74 and 8.62 days. When *c *is smaller than 1 (i.e. when there are more asymptomatic infected individuals with extremely long generation times compared to symptomatic cases), the effectiveness measure (A9) becomes smaller than the baseline which we get from (4) in the main text. On the contrary, if the generation time of asymptomatic infected individuals is shorter than that of symptomatic infected individuals, the effectiveness rises up close to 100% with the assumed lengths of quarantine, suggesting the need to accumulate epidemiological evidence of the generation time.

Second, we investigate the impact of differing sensitivity of rapid diagnostic testing between symptomatic and asymptomatic cases on the effectiveness of quarantine. We theoretically separate the sensitivity S_e _into S_e, s _and S_e, a _for symptomatic and asymptomatic cases, respectively. Since asymptomatic cases may shed lower titres of virus, we suspect that the ratio S_e, a _to S_e, s _(*r *:= S_e, a_/S_e, s_) is smaller than 1. The equation (5) in the main text is replaced by

(A10) *ε*_*d*_(*t*) = 1- [(1 - S_e, s_) *α*(1 - *F*(*t*))(1 - *G*(*t*)) + (1 - S_e, a_) (1 - *α*)(1 - *G*(*t*))]

Figure 7C shows the sensitivity of *ε*_d_(*t*) to different values of the ratio *r *assuming that S_e, s _= 0.69. As the ratio *r *becomes smaller (i.e. as the diagnosis of asymptomatic infected individuals becomes more difficult than that of symptomatic cases), the effectiveness also becomes smaller. Although the difference in *ε*_d_(*t*) is greater for short quarantine periods, the effectiveness becomes less sensitive to *r *as the length of quarantine becomes longer. We estimated that 99.0% effectiveness in reducing the risk of introducing infectious individuals into the community is achieved with *t *= 5.71 days using the rapid diagnostic test of *r *= 1.0 in the main text. The effectiveness estimate with the same length of quarantine and *r *= 0.6 is still as large as 98.1%.

### Imperfect case detection

Although we considered perfect detection of symptomatic cases upon symptom onset during quarantine in the main text, here we examine the sensitivity of the effectiveness of quarantine to differing efficacy of case detection. Let the efficacy of case finding be *k *which we assumed as 1 in the main text. In reality, it might be difficult to detect all flu-like symptoms (i.e. *k *< 1). The equation (4) in the main text is replaced by

(A11) *ε*(*t*) = 1 - [*α*(1 - *kF*(*t*)) (1 - *G*(*t*)) + (1 - *α*) (1 - *G*(*t*))]

It should be noted that *k *influences symptomatic cases alone, because the detection of symptoms does not apply to asymptomatic infected individuals. Figure 7D shows the sensitivity of *ε *(*t*) to different values of the ratio *k *which was assumed to lie in the range of 0.6 – 1.0. As the ratio *k *becomes smaller (i.e. as the detection becomes less efficient), the effectiveness becomes smaller. The difference in *ε *(*t*) between different ratios *k *is particularly highlighted when the quarantine period is between 2 and 5 days. Nevertheless, for the shorter and longer quarantine periods, difference in *ε *(*t*) is almost negligible. In the main text, we estimated that quarantine for 8.62 days achieves 99.0% effectiveness of reducing the risk of releasing infectious individuals into the community with *k *= 1.0. The effectiveness estimate with the same length of quarantine and *k *= 0.6 is still as large as 98.2%.

## Pre-publication history

The pre-publication history for this paper can be accessed here:

http://www.biomedcentral.com/1471-2334/9/27/prepub
